# Investigation of antibacterial properties silver nanoparticles prepared via green method

**DOI:** 10.1186/1752-153X-6-73

**Published:** 2012-07-27

**Authors:** Kamyar Shameli, Mansor Bin Ahmad, Seyed Davoud Jazayeri, Parvaneh Shabanzadeh, Parvanh Sangpour, Hossein Jahangirian, Yadollah Gharayebi

**Affiliations:** 1 Department of Chemistry, Faculty of Science, Universiti Putra Malaysia, 43400, UPM Serdang, Selangor, Malaysia; 2 Materials & Energy Research Center, Alborz, Karaj, P.O. Box: 31787/316, Iran; 3 Institute of BioSciences, Universiti Putra Malaysia, 43400, Serdang, Selangor Darul Ehsan, Malaysia; 4 Department of Chemical Engineering, Faculty of Engineering, Islamic Azad University, Malard Branch, Iran; 5 Department of Chemistry, Islamic Azad University Behbahan Branch, University Street, Behbahan, 6361713198, Iran

**Keywords:** Silver nanoparticles, Green chemistry, Polyethylene glycol, Antibacterial activity, Reaction time effect

## Abstract

**Background:**

This study aims to investigate the influence of different stirring times on antibacterial activity of silver nanoparticles in polyethylene glycol (PEG) suspension. The silver nanoparticles (Ag-NPs) were prepared by green synthesis method using green agents, polyethylene glycol (PEG) under moderate temperature at different stirring times. Silver nitrate (AgNO_3_) was taken as the metal precursor while PEG was used as the solid support and polymeric stabilizer. The antibacterial activity of different sizes of nanosilver was investigated against Gram–positive [*Staphylococcus aureus*] and Gram–negative bacteria [*Salmonella typhimurium SL1344*] by the disk diffusion method using Müeller–Hinton Agar.

**Results:**

Formation of Ag-NPs was determined by UV–vis spectroscopy where surface plasmon absorption maxima can be observed at 412–437 nm from the UV–vis spectrum. The synthesized nanoparticles were also characterized by X-ray diffraction (XRD). The peaks in the XRD pattern confirmed that the Ag-NPs possessed a face-centered cubic and peaks of contaminated crystalline phases were unable to be located. Transmission electron microscopy (TEM) revealed that Ag-NPs synthesized were in spherical shape. The optimum stirring time to synthesize smallest particle size was 6 hours with mean diameter of 11.23 nm. Zeta potential results indicate that the stability of the Ag-NPs is increases at the 6 h stirring time of reaction. The Fourier transform infrared (FT-IR) spectrum suggested the complexation present between PEG and Ag-NPs. The Ag-NPs in PEG were effective against all bacteria tested. Higher antibacterial activity was observed for Ag-NPs with smaller size. These suggest that Ag-NPs can be employed as an effective bacteria inhibitor and can be applied in medical field.

**Conclusions:**

Ag-NPs were successfully synthesized in PEG suspension under moderate temperature at different stirring times. The study clearly showed that the Ag-NPs with different stirring times exhibit inhibition towards the tested gram-positive and gram-negative bacteria.

## Background

Silver nanoparticles (Ag-NPs) have been known for its inhibitory and bactericidal effects in the past decades [1]. Antibacterial activity of silver containing materials can be applied in medicine for reduction of infections on the burn treatment [2,3], prevention of bacteria colonization on catheters [4,5] and elimination of microorganisms on textile fabrics [6,7] as well as disinfection in water treatment [8]. Besides that, Ag-NPs were also being reported in the literature to exhibit a strong cytoprotective activity towards human immunodeficiency virus (HIV) infections [9]. Polyethylene glycol (PEG) is frequently used in the polymer blends production to improve the biocompatibility of its film due to its wide range of molecular weights, excellent solubility in aqueous medium, low toxicity, chain flexibility and biocompatibility properties. Although PEG has non biodegradability properties, it is readily excreted from the body and forms non-toxic metabolites [10]. Besides that, PEG was able to act both as reducing agent and stabilizer [11]. In several research studies [12,13], researchers proposed that longer polymer chain of PEG exhibits higher reducing activity and provides higher stability in forming Ag-NPs. These can effectively prevent agglomeration of Ag-NPs.

There are numerous techniques to perform antibacterial and antimicrobial susceptibility tests. The techniques include agar disk diffusion, broth dilution (macrodilution and microdilution), agar dilution and E test method (modification of the disk diffusion and the agar dilution method) [14]. Agar disk diffusion is a traditional and routine method for antimicrobial susceptibility tests [15]. It has advancement to be used in this project because of its reliability, low cost and simplicity [16,17]. Mueller Hinton agar is chosen among the culture media because it gives satisfactory growth for most nonfastidious organisms like *Staphylococcus aureus*, *Pseudomonas aeruginosa* and *Escherichia coli* and it shows good bacteria culture reproducibility [18].

There are many synthetic routes that have been developed to synthesize Ag-NPs due to the applications found tremendously in wide range of fields. Among the synthetic routes includes chemical reduction [19,20], thermal decomposition [21], electrochemical [22], sonochemical [23], photochemical [24], microwave [25], radiation assisted process [26,27] and currently by green chemistry synthesis [28-31].

Chemical reduction method is widely used to synthesize Ag-NPs because of its readiness to generate Ag-NPs under gentle conditions and its ability to synthesize Ag-NPs on a large scale [32]. However, these chemical synthesis methods employ toxic chemicals in the synthesis route which may have adverse effect in the medical applications and hazard to environment. Therefore, preparation of Ag-NPs by green synthesis approach has advantages over physical and chemical approaches as it is environmental friendly, cost effective and the most significant advantage is that conditions of high temperature, pressure, energy and toxic chemicals are not required in the synthesis protocol [33].

In this work, we reported “green” synthesis of Ag-NPs using sugar and PEG. This method was performed by reducing the silver nitrate (AgNO_3_) in different stirring times of reaction at moderate temperature with sugar and PEG used as green reducing agent and polymeric stabilizer. The antibacterial activity of silver/polyethylene glycol [Ag(PEG)] were tested with Mueller-Hinton agar disc diffusion method against *Staphylococcus aureus* (*S. aureus*), and *Salmonella typhimurium SL1344* (*S*. *typhimurium SL1344*).

## Results and discussion

**Figure 1:**
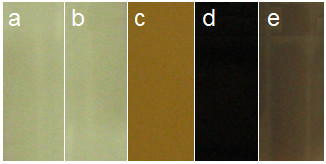
Photograph of Ag-NPs prepared at different times of reaction in PEG solution in the moderate temperatures for 1, 3, 6, 24 and 48 h (a–e), respectively.

In this research, the PEG was appropriate as a stabilizer and polymeric media for reducing the AgNO_3_ using sugar as a green reducing agent. As shown in Figure 1, after 1 and 3 h, the colourless solution turned to yellow which indicates the initial formation of Ag-NPs. Similarly, when the time of reaction was increased to about 6 h, the colour changed to the light brown. However, when the solution was further stirred for a period of 48 h at a temperature of 25, the colour of the solution change to dark brown and then gray. These observations show that with the increase time of reaction, particle size and aggregation of silver nanocrystal gradually increased.

Sugar as an aldehyde can reduce silver ions to Ag-NPs and through this process oxidizes itself gluconic acid [34]. The possible chemical equations for preparing the Ag-NPs are:

(1) $$\;{\text{Ag}}_{\left(\text{aq}\right)}^{+}+{\text{PEG}}_{\left(\text{aq}\right)}\to {\left[\text{Ag}(\text{PEG})\right]}_{\text{aq}}^{+}$$

(2) $$\;\begin{array}{c} 2{\left[\text{Ag}\left(\text{PEG}\right)\right]}_{\text{aq}}^{+}+{\text{CH}}_{2}\text{OH}{\left(\text{CHOH}\right)}_{4}\text{CHO}\\ \;\to 2[\text{Ag}\left(\text{PEG}\right)]↓+{\text{CH}}_{2}\text{OH}{\left(\text{CHOH}\right)}_{4}\text{COOH} \end{array}$$

After dispersion of silver ions in the PEG aqueous solution matrix (Equation 1), PEG reacted with the Ag to form a PEG complex [Ag(PEG]^+^, which reacted with sugar to form [Ag(PEG)] due to the reduction of silver ions through the oxidation of sugar to gluconic acid (Equation 2).

### UV–visible spectroscopy

**Figure 2:**
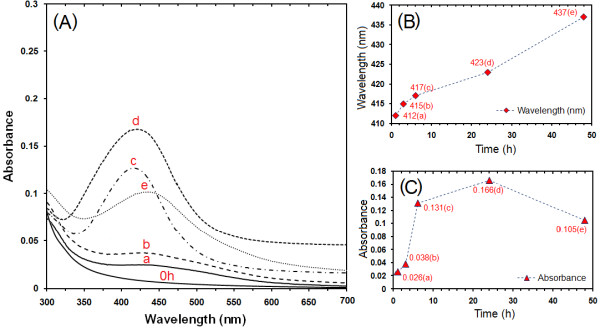
The Ultraviolet–visible spectra curve of Ag-NPs prepared in PEG solution at different times in the moderate temperatures (A–C).

The formation of Ag-NPs in the polymeric media was further determined by using the UV–visible spectroscopy, which was shown on the surface plasmon resonance (SPR) bands. Figure 2 (A–C) shows that Ag-NPs started forming when [Ag(PEG)]^+^ reacted with suger at a moderate temperature. However, the [Ag(PEG)]^+^ peak was not observed at beginning (0 h) of the reaction until after about 1 h of the reaction time, the absorbance peaks could be seen at different stirring times after the reaction started. Generally, the SPR bands are influenced by the size, shape, morphology, composition and dielectric environment of the prepared nanoparticles [35,36]. Previous studies have shown that the spherical Ag-NPs contribute to the absorption bands at around 400 nm in the UV–visible spectra [37]. From this research, the SPR band characteristics of Ag-NPs were detected around 412–437 nm (Figure 2A, B), which strongly suggests that the Ag-NPs were spherical in shape and have been confirmed by the TEM results of this study. As shown, when the stirring time of reaction was increased, the intensity of the SPR peak also gradual increase until 24 h but after 48 h the SPR peak change to broad shape and intensity decreased, this phenomenon is related to the increased size and also agglomeration of silver nano-crystals [38]. Therefore this shows that the reduction of the silver ions to silver atoms continued and resulted in an increase in the concentration of Ag-NPs [39].

Thus, there is a normal case in this situation for the SPR absorption band for the particles, which agreed with the TEM results, whereby red–shifts were observed as size increased in the during the reaction after 1, 3, 6, 24 and 48 h respectively. This can be explained by the multilayer Mie theory model, which theorizes that the chemical interaction caused the lowered electron conductivity in the outermost atomic layer and consequently caused the red–shifts [40]. As seen from the Figure 2C, it can be observed that 24 h had large absorbance compared to 48 h because the particle size of Ag-NPs after 48 h were larger than those at 24 h. Also, absorption spectra of larger metal colloidal dispersions can exhibit broad peaks or additional bands with the lower absorbance in the UV-visible range due to the excitation of plasma resonances or higher multipole plasmon excitation [41]. This phenomenon could be due to the fact that, after reaching a certain particle size, the stabilizer was not able to withhold the nanoparticle’s size effectively, which resulted in its very large size.

### Morphology study

**Figure 3:**
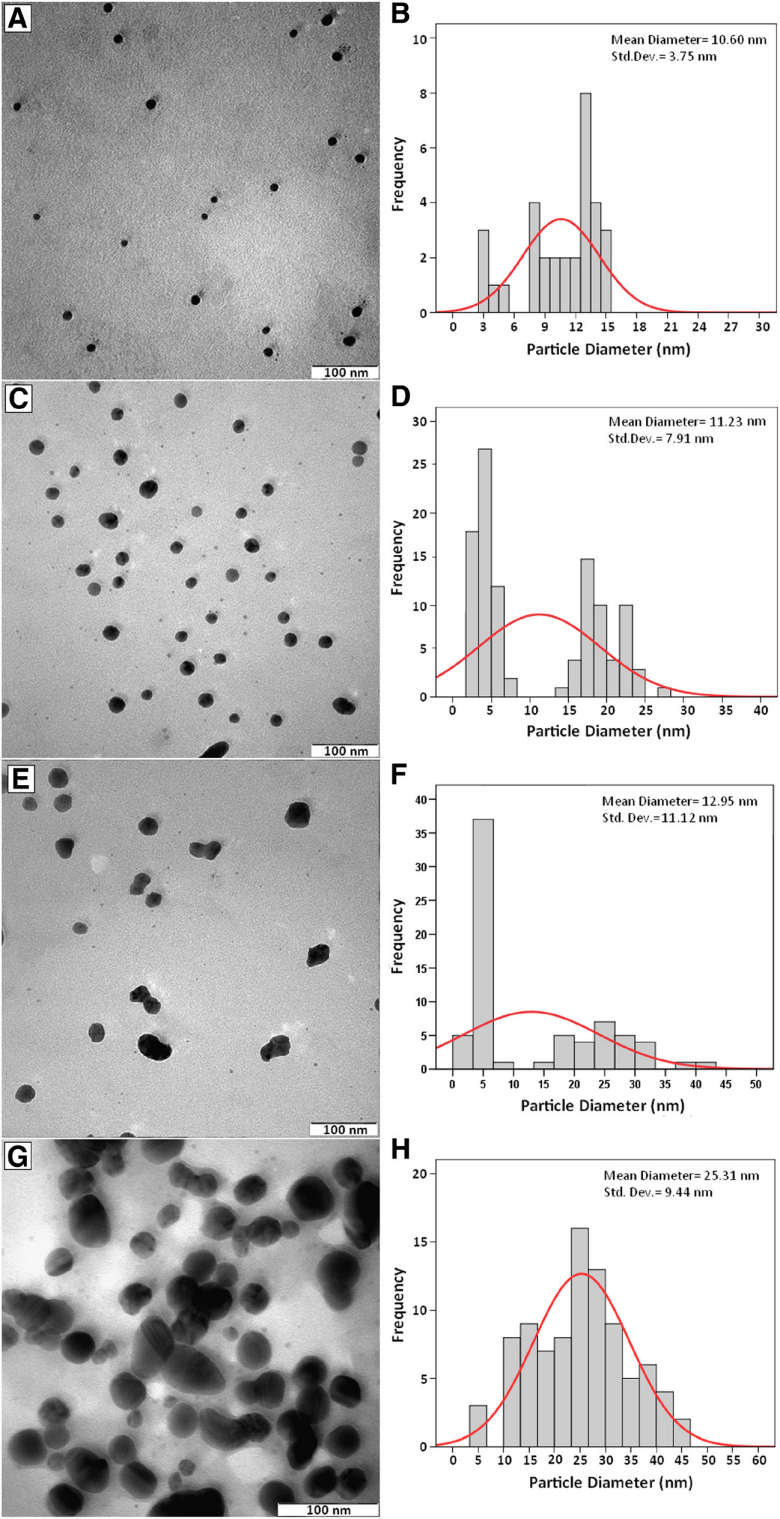
Transmission electron microscopy image and the particle size distribution for Ag-NPs in PEG for the stirring times of 3, 6, 24 and 48 h, respectively (A–D).

The TEM images and their corresponding particle size distributions of Ag-NPs at different periods of time are shown in Figure 3. The TEM images and their size distributions revealed that, the mean diameters and standard deviation of Ag-NPs were about 10.60 ± 3.75, 11.23 ± 7.91, 12.95 ± 11.12 and 25.31 ± 9.44 nm for 3, 6, 24 and 48 h (A–D), respectively. The total numbers of Ag-NPs counted for each TEM images were about 32, 107, 226 and 64 for 3, 6, 24 and 48 h respectively. These results approved that with increase in time of reaction at a moderate temperature, mean diameters and standard deviations of the Ag-NPs gradually increases.

### Powder X–ray diffraction

**Figure 4:**
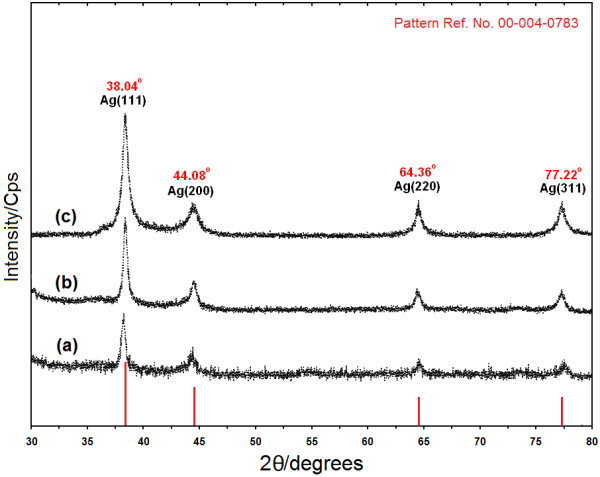
X-ray diffraction patterns of Ag-NPs synthesized in PEG after 6, 24 and 48 h respectively (a–c).

Figure 4 shows the XRD patterns of Ag-NPs formed in the 6 h, 24 h, and 48 h from stirring time of reaction, which indicates the formation of the silver crystalline structure. The XRD peaks in the wide angle range of 2θ (30° < 2θ < 80°) ascertained that the peaks in 38.04°, 44.08°, 64.36° and 77.22° can be attributed to the 111, 200, 220, and 311 crystalline structures of the face centered cubic (fcc) synthesized silver nano–crystal, respectively (Ag XRD Ref. No. 00–004–0783) [42]. The intensities of 111, 200, 220 and 311 reflections due to the Ag-NPs phase were also found to increase along with the increased Ag-NPs in the polymeric media (Figure 4a–c). The peaks showed that the main composition of nanoparticles was silver and clearly no obvious other peaks present as impurities were found in the XRD patterns. Therefore, this gives clear evidence for the presence of Ag-NPs in the [Ag (PEG)].

The average particle size of silver nanoparticles can be calculated using Debye–Scherrer equation (3):

(3) $$\;n=\frac{K\lambda }{\beta cos\theta }$$

Where K is the Scherrer constant with value from 0.9 to 1 (shape factor), where *λ* is the X-ray wavelength (1.5418 Å), β_1/2_ is the width of the XRD peak at half height and *θ* is the Bragg angle. From the Scherrer equation the average crystallite size of Ag-NPs for 6, 24 and 48 h times of reaction are found to be around 10–25 nm, which are also in line with the observation of the TEM results discussed later.

### Zeta potential measurement

**Figure 5:**
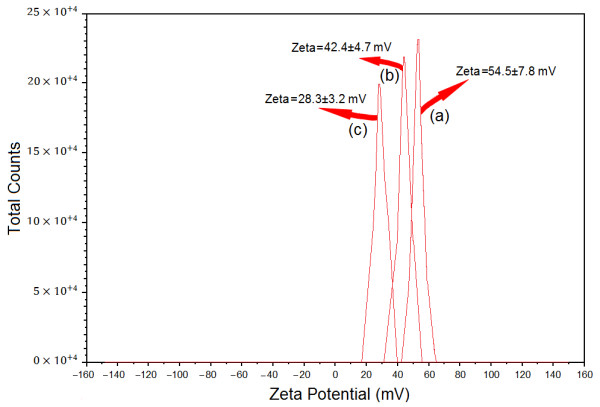
Zeta potential for [Ag (PEG)] suspension after 6, 24 and 48 h from stirring times (a–c).

As shown in the Figure 5, the Ag-NPs obtained possess a positive zeta potential value. Zeta potential is an essential parameter for characterization of stability in aqueous Ag-NPs suspensions. A minimum of ±30 mV zeta potential values is required for indication of stable nano-suspension [43]. At the 6, 24 and 48 h of stirring times zeta potential were equals to 54.5 ± 7.8, 42.4 ± 4.7 and 28.3 ± 3.2 mV respectively. So, these results clearly indicates that the particles are fairly stable at the 6 h stirring time of reaction, but the stability decreased when the reaction time was increased to 24 h and 48 h respectively.

### FT-IR chemical analysis

**Figure 6:**
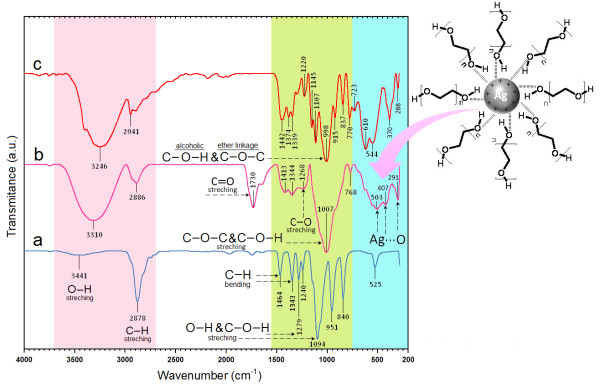
Fourier transform infrared spectra for PEG (a), [Ag (PEG)] after 48 h (b) from stirring times and sugar (c).

The interaction of Ag-NPs obtained with PEG and gluconic acid products by reduction of sugar compound were confirmed by FT-IR spectra. Intense absorptions are observed at 1730, 1630 and 1007 cm^−1^. The IR band at 1730 cm^−1^ is characteristic of the C = O stretching mode of the carboxylic acid group for gluconic acid. The bands due to C–O stretching mode got merged in the very broad envelope centered on 1268 and 1007 cm^−1^ arising from C–O, C–O–C stretches and C–O–H bends vibrations of Ag-NPs in PEG. Also, the aliphatic C–H stretching, in 1413 and 1344 cm^−1^ were due to C–H bending vibrations (Figure 6a) [44]. After the bio-reaction of sugar with the AgNO_3_ in the PEG matrix, the created peak in 1730 cm^−1^ certified to the binding of –C = O for carboxylic acid in gluconic acid, and the shift in the peak at 1007 cm^−1^ towards lower frequency compared to peak in 1094 cm^−1^ for PEG is attributed to the binding of C–C–O and C–C–H groups with nanoparticles [45]. The broad peaks in 503, 407 and 291 cm^−1^ are related to Ag-NPs banding with oxygen from hydroxyl groups of PEG chains (Figure 6b). On the other hand, as for the sugar spectrum (Figure 6c), the absorption bands at 3246 cm^−1^ was due to the O–H stretching band, 2901 cm^−1^ was due to the aliphatic C–H stretching, 1442, 1374 and 1339 cm^−1^ were due to C–H bending vibrations, and also the combination band of O–C–H and C–O–H deformation is calculated from 1442 to 1339 cm^−1^. Then the plane C–H and O–H deformation from 1220 to 998 cm^−1^ can be observed. The region from 1145 to 554 cm^−1^ contains C–O and C–C groups’ vibration modes are present and the carbohydrates generally shows their characteristic bands [46].

Thus, as shown hydroxyl group of PEG as capping agent can make a cover in the surface of Ag-NPs. This is possible because the surface of Ag-NPs is positively charged. Certainly, we suppose that colloidal stabilization for [Ag (PEG)] occur due to the presence of van der waals forces between the oxygen negatively charged groups present in the molecular structure of the PEG, and the positively charged that surround the surface of the inert Ag-NPs [45,47]. Therefore, the FT-IR spectra showed the existence molecular interactions between the Ag-NPs with the chain of polymeric media [47]. As shown in the Figure 6, schematic illustrated the interaction between the charged of Ag-NPs that capped with PEG [28,48].

### Antibacterial activity

**Figure 7:**
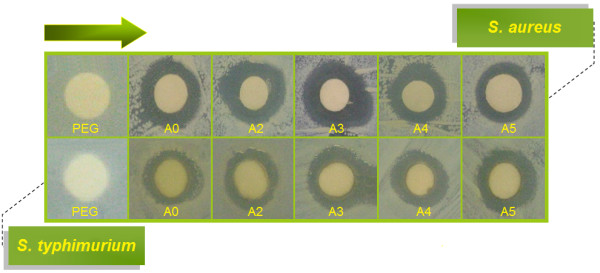
**Comparison of the inhibition zone test between Gram-positive and Gram-negative bacteria [*S. aureus *and *S*. *typhimurium*] for PEG, [Ag (PEG)]**^**+**^**(A0) and [Ag (PEG)] suspension at different stirring times [A2–A5 (3, 6, 24 and 48 h)].**

Inhibition zone values were obtained for PEG, [Ag(PEG)]^+^ (A0) and [Ag (PEG)] suspension at the different stirring times 3 (A2), 6 (A3), 24 (A4) and 48 h (A5) and tested against *S. aureus* and *S*. *typhimurium*. The results and images of inhibition zones are presented as the average values in Table 1 and Figure 7, respectively. Table 1 shows that the AgNO_3_ and Ag-NPs in PEG suspension gave high and similar antibacterial activity against Gram-negative and Gram-positive bacteria. Because of their size, Ag-NPs can easily reach the nuclear content of bacteria and they present the large and impressive surface area; thus, the contacts with bacteria were the greatest [49,50]. This could be the reason behind their excellent antibacterial effect. In the polymeric matrix systems, some researcher argue that silver ions released from the surface of Ag-NPs are responsible for their antibacterial activity [51,52]. In the aqueous phase systems, the results show that the antibacterial activity of Ag-NPs at 3 and 6 h stirring times in *S.aureus* is higher than that of the Ag^+^ ions. Similarly, the antibacterial activity of Ag-NPs in *S. typhimurium* is generally higher than that of the Ag^+^ ions. With the exception of the Ag-NPs at 3 h stirring time, the activity decreased with increase in stirring time (6, 24 and 48 h). The high activity at the 6 h stirring time Ag-NPs is perhaps related to large surface area of the nanoparticles [53]. The diameters of inhibition zone in the agar plate are given in mm. The tests were replicated three times for each treated samples and the results are presented in Table 1. The solution of PEG (10 mg/ml) did not show any antibacterial activity. The [Ag(PEG)]^+^ (A0) suspension for all tested bacteria shows high antibacterial activity and interestingly these effects in the [Ag (PEG)] (A2–A5) were increased with the decreasing size of Ag-NPs. However, a higher Ag-NPs loading doesn’t improve the antibacterial activity [54].

**Table 1:** **Average inhibition zone and standard deviation for PEG, [Ag (PEG)]**^**+**^**(A0) and [Ag (PEG)] suspension (A2–A5) at different stirring times (3, 6, 24 and 48 h), respectively**

**Bacteria**	**Inhibition zone(mm)**					**Control -**	**Control +**	
	**A0**	**A2**	**A3**	**A4**	**A5**	**(mm)**	**(mm)**	
						**PEG**	**CTX**	**C**
*S.aureus*	11.65 ± 0.56	12.78 ± 0.12	13.64 ± 0.29	11.56 ±0.36	9.71 ± 0.14	NA	18.45	17.45
*S. typhimurium*	9.67 ± 0.33	9.44 ± 0.36	11.51 ± 0.43	10.64 ± 0.39	10.62 ± 0.36	NA	18.75	16.58

Abbriviation: NA, Not Appear; CTX, Chloramphenicol; C, Cefotaxime.

## Conclusions

In summary, we have described a simple and green method of colloidal Ag-NPs synthesis by using green reducing agents which requires no special physical conditions. Ag-NPs were successfully synthesized under moderate temperature (45°C) at different stirring times of reaction. The formation of Ag-NPs was confirmed in the UV-visible absorption spectra, which showed the SPR band characteristics of Ag-NPs in the range of 412–437 nm. The XRD results confirmed that the Ag-NPs possessed a face-centered cubic crystal structure (fcc). In addition, this also revealed that Ag-NPs were the main composition present in the nanocomposites without any contamination peaks. The TEM images showed that the Ag-NPs were in spherical shape and the average diameters of the particles were 10.60, 11.23, 12.95 and 25.31 nm for the stirring times of 3, 6, 24 and 48 h, respectively. FTIR spectrum suggested the complexation present between PEG and Ag-NPs to form metallopolymer [Ag (PEG)] and the stability of the Ag-NPs was confirmed with the zeta potential measurements. The antibacterial activities of [Ag (PEG)] at the different particle size of Ag-NPs were showed antibacterial activity against the Gram-positive and Gram-negative bacteria. These results show that the antibacterial activities of Ag-NPs in PEG can be modified with the size of Ag-NPs and it decreases with the increase in the particle size. Needless to say, further studies are required to investigate the biological effects of [Ag (PEG)] suspension on the types of bacteria for potential widening of this subject area.

## Methods

### Materials

All reagents in this effort were analytical grade and were used as received without further purification. AgNO_3_ (99.98%) was used as a silver precursor, and was provided by Merck, Germany. PEG (*Mw 3,350*) used as a stabilizer for the preparation of Ag-NPs which was purchased from Sigma–Aldrich (USA). Meanwhile, the sugar was used as a green reducing of silver ions to Ag atoms and was obtained from BDH Chemical Ltd., Poole, UK. All solutions were freshly prepared using double distilled water and kept in the dark to avoid any photochemical reactions. All glassware used in experimental procedures were cleaned in a fresh solution of HNO_3_/HCl (3:1, v/v), washed thoroughly with double distilled water, and dried before use.

### Synthesis of Ag-NPs by using green method

The preparation of Ag-NPs in the PEG matrix is quite directly forward. In a typical synthesis, a 10 mL of a 1.0 M solution of AgNO_3_ was added to 200 mL of a 0.1 wt.% aqueous solution of soluble PEG to obtain the clear solution ${\left[\text{Ag}\left(\text{PEG}\right)\right]}_{\text{aq}}^{+}$. After complete dissolution of these components, 20 mL of a 1.0 M aqueous solution of sugar was then added and further stirred. The solution obtained was distributed into 5 cuvettes, which were stirred and maintained at different periods of time: 1 (a), 3 (b), 6 (c), 24 (d), and 48 h (e), respectively. Throughout the reduction process, all solutions were kept at a constant temperature of 60°C in the dark to avoid any photochemical reactions. All solution components were purged with nitrogen gas prior to use. Subsequently, reduction proceeded in the presence of nitrogen to eliminate oxygen. The obtained colloidal suspensions of [Ag (PEG)] were then centrifuged at 20000 rpm for 15 min and washed four times to remove silver ion residue. The precipitate nanoparticles were then dried overnight at 40°C under vacuum overnight to obtain the Ag-NPs.

### Evolution of antibacterial activity

The in vitro antibacterial activity of the samples was evaluated by utilizing the disc diffusion method using Müeller–Hinton Agar (MHA) with determination of inhibition zones in millimeter (mm), which conform with recommended standards of the National Committee for Clinical Laboratory Standards (NCCLS; now renamed as Clinical and Laboratory Standards Institute, CLSI, 2000). *Salmonella typhimurium* (*S*. *typhimurium* SL1344) and *Staphylococcus aureus* (*S. aureus*) (ATCC 25923) were used for the antibacterial effect assay. Briefly, the sterile paper discs (6 mm) impregnated with 20 μl of PEG, ${\left[\text{Ag}\left(\text{PEG}\right)\right]}_{\text{aq}}^{+}$ and [Ag (PEG)] (3, 6, 24 and 48 h) with different treatment times were suspended in sterile distilled water and were left to dry at 37°C for 24 h in a sterile condition. The bacterial suspension was prepared by making a saline suspension of isolated colonies selected from tryptic soy agar plate, the agar plates were grown for 18 to 24 h. The suspension was adjusted to match the tube of 0.5 McFarland turbidity standard using the spectrophotometer of 600 nm, which equals to 1.5 × 10^8^ colony–forming units (CFU)/ml. The surface of MHA was completely inoculated using a sterile swab, which steeped in the prepared suspension of bacterium. Finally, the impregnated discs were placed on the inoculated agar and incubated at 37°C for 24 h. After incubation, the diameter of the growth inhibition zones was measured. Chloramphenicol (30 μg) and Cefotaxime (30 μg) were used as the positive standards in order to control the sensitivity of the bacteria. All tests were done in triplicate.

### Characterization methods and instruments

The prepared Ag-NPs were characterized by using the X–ray diffraction (XRD), transmission electron microscopy (TEM), ultraviolet–visible spectroscopy, Fourier transform infrared (FT–IR) spectroscopy and zeta potential measurements. The XRD patterns were recorded at a scan speed of 2° min–^1^. Meanwhile, the structures of the produced Ag-NPs were examined using Shimadzu PXRD–6000, powder X–ray diffraction. Moreover, TEM observations were carried out using the Hitachi H–7100 electron microscopy, whereas the particle size distributions were determined using the UTHSCSA Image Tool software (Version 3.00). To make sure the formation of Ag-NPs, the colloids solutions were tested for their optical absorption property using a Shimadzu H.UV, 1650 PC UV–visible spectrophotometer over the range of 300 to 700 nm. The FT–IR spectra were however recorded over the range of 200–4000 cm^−1^ utilizing the Series 100 Perkin Elmer FT–IR 1650 spectrophotometer. The zeta potential measurements were also performed using a Zetasizer Nano–ZS (Malvern Instruments).

## Competing interests

We declare that we have no competing interests.

## Authors’ contributions

KS carried out the synthesis, and characterization of the compounds, acquisition of data, analysis and interpretation of data collected. SDJ carried out the antibacterial experiments. MBA and PS involved in drafting of manuscript, revision of draft for important intellectual content and give final approval of the version to be published. All authors read and approved the final manuscript.
